# Comparison of pain levels in fusion prostate biopsy and standard TRUS-Guided biopsy

**DOI:** 10.1590/S1677-5538.IBJU.2019.0154

**Published:** 2020-03-12

**Authors:** Abdullah Demirtaş, Gökhan Sönmez, Şevket Tolga Tombul, Türev Demirtaş

**Affiliations:** 1 Department of Urology Erciyes University Kayseri Turkey Department of Urology, Erciyes University, Kayseri, Turkey;; 2 Department of Urology Kayseri City Hospital Kayseri Turkey Department of Urology, Kayseri City Hospital, Kayseri, Turkey;; 3 Department of Medical History and Ethics Erciyes University Kayseri Turkey Department of Medical History and Ethics, Erciyes University, Kayseri, Turkey

**Keywords:** Prostate, Biopsy, Pain

## Abstract

**Objectives:**

Fusion prostate biopsy (FPB) has recently emerged as a popular and successful biopsy technique on diagnosis of prostate cancer. The aim of this study was to compare the pain levels in TRUS-guided standard 12-core prostate biopsy (SPB) and MpMRI-guided FPB.

**Materials and Methods:**

Patients detected with a PI-RADS (Prostate Imaging Reporting and Data System) ≥3 lesion on MpMRI underwent MpMRI-guided FPB (Group I) and the patients who had no suspected lesions or had a PI-RADS <3 lesion on MpMRI underwent TRUS-guided SPB (Group II). Pain assessment was performed using Visual Analog Scale (VAS) five minutes after the procedure. Following the procedure, the patients were asked to indicate the most painful biopsy step among the three steps.

**Results:**

252 patients were included in this study (Group I=159, Group II=93). The mean number of cores and the malignancy detection rate were significantly higher in Group I compared to Group II (p <0.001, p=0.043, respectively). No significant difference was found between the two groups with regard to VAS scores (p=0.070). The most painful part of the whole procedure was revealed to be the insertion of the probe into the rectum. However, no significant difference was found between the two groups with regard to the most painful biopsy step (p=0.140).

**Conclusion:**

FPB, with a relatively higher cancer detection rate, leads to the same pain level as SPB although it increases the number of biopsy cores and involves a more complex procedure compared to SPB. Further prospective studies with larger patient series are needed to substantiate our findings.

## INTRODUCTION

Prostate cancer is the second most common cancer in men ( 1 ). Common procedures performed in the treatment of prostate cancer include digital rectal examination (DRE) and the serum prostate-specific antigen (PSA) test ( 2 ).

Periprostatic nerve block (PNB) is one of the most common and effective anesthetic techniques used for pain management during transrectal ultrasound (TRUS)-guided standard 12-core prostate biopsy (SPB) ( 3 , 4 ). Additionally, intrarectal administration of anesthetic drugs has also been shown to reduce the pain level during biopsy ( 5 , 6 ). In the past, prostate biopsies performed without these methods had caused serious difficulties for clinicians and patients. Although analgesia and anesthesia methods used today decreased pain significantly, patients continue to experience some pain ( 7 , 8 ).

Multiparametric magnetic resonance imaging (MpMRI)-guided fusion prostate biopsy (FPB) has recently emerged as a popular technique with the aid of technological advancements. Despite involving a more complex procedure compared to SPB, this technique has been shown to provide successful outcomes in numerous studies ( 9 - 11 ). In this technique, unlike in SPB, additional biopsy cores beside 12 biopsy cores are obtained from the suspicious lesions detected on MpMRI ( 12 ).

In this study, we aimed to compare the pain levels in the patients that underwent TRUS-guided SPB and MpMRI-guided FPB in our clinic.

## MATERIALS AND METHODS

### Patients

This prospective study included patients that underwent prostate biopsy due to suspected prostate cancer at Department of Urology, Erciyes University, between December 2016 and January 2019. Patients detected with a PI-RADS (Prostate Imaging Reporting and Data System) ≥3 lesion on MpMRI underwent MpMRI-guided FPB (Group I) and the patients who had no suspected lesions or had a PI-RADS <3 lesion on MpMRI underwent TRUS-guided SPB (Group II). Patients with a previous negative biopsy, neurological disorders that could affect the pain level such as paraplegia or hemiplegia, and a serum total PSA level of >50ng/mL were excluded from the study. Moreover, patients that used analgesics for any reason on the day of or the day before the procedure, underwent biopsy under general anesthesia, and had such diseases as anal fissure or hemorrhoidal disease that could alter the pain threshold were also excluded from the study.

### Pre-biopsy procedure

Appropriate antibiotic prophylaxis was performed in each patient based on the results of rectal swabbing administered before the biopsy procedure. No bowel preparation or rectal cleansing was administered prior to the procedure. A MpMRI scan was performed without an endorectal coil in each patient (Siemens, Magnetom, 1.5 T).

### Local anesthesia

Initially, 2% lidocaine gel was applied to the anal cavity of each patient. After waiting for 10 minutes, the ultrasound probe was introduced into the rectum and PNB was performed by infiltrating 5mL 2% prilocaine (VEM Medicine, Istanbul, Turkey) diluted 1:1 into the angle between the seminal vesicle and base of the prostate in the parasagittal plane with an 18-gauge (G) and 30-centimeter (cm) needle.

### Biopsy procedure and pain assessment

TRUS-guided SPB was performed by obtaining 10-12 core samples in each patient. FPB was performed by obtaining 10-12 core samples in each patient, followed by the acquisition of 2-4 core samples for each suspected lesion detected on MpMRI (combined biopsy). All the biopsy procedures were performed using an ultrasound (US) fusion device based on rigid registration (Logiq E9, GE, USA) with an endorectal single-angle probe (type: IC5-9-D).

**Figure 1:**
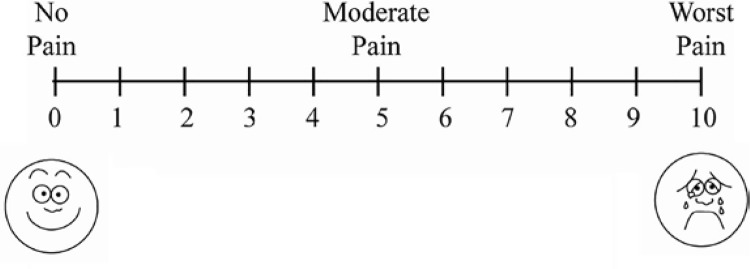
Visual Analog Scale.

Prior to the biopsy procedure, each patient was verbally informed about the three biopsy steps (1: insertion of the probe into the rectum, 2: probe manipulation, and 3: the piercing of the biopsy needle) and were asked to indicate the most painful step for them after the procedure. Following the procedure, pain assessment was performed using Visual Analogue Scale (VAS) ( 13 ). VAS is a self-reporting measure of pain intensity consisting of a 0-10 scale, whereby 0 indicates no pain and 10 indicates the most severe and intolerable pain ( Figure-1 ).

### Data collection and statistical analysis

Patient data regarding age (years), body mass index (BMI), serum PSA levels, prostate volumes measured during the procedure, total number of cores obtained by biopsy, VAS scores, the most painful biopsy step, and histopathological examination results were recorded for each patient. Prostate volume was measured following the administration of PNB using the following formula: Height x Width x Length x 0.523.

Statistical analyses were performed using IBM SPSS 22.0 (Armonk, NY: IBM Corp.). Normal distribution of data was analyzed using Kolmogorov-Smirnov and Shapiro-Wilk tests. Descriptive data were expressed as mean±standard deviation (SD) or median (25th-75th percentile) based on the distribution pattern of the data. Variables with normal distribution were compared using Independent Samples t-test. Quantitative variables with non-normal distribution and independent groups with ordinal data were compared using Mann-Whitney U test. Categorical variables were compared using Chi-square test. A p value of <0.05 was considered significant.

### Ethical approval

In this study, written and verbal consent was obtained from the patients for biopsy and study procedures. The study was approved by the Erciyes University Clinical Research Ethics Committee (Approval No. 2014-508).

## RESULTS

The study included a total of 252 patients, comprising 159 (63.1%) patients that underwent FPB (Group I) and 93 (36.9%) patients that underwent SPB (Group II). Mean age was 61.99 (±6.95) years, median BMI was 26.10 (22.80-28.20) kg/m^2^, median serum PSA level was 7.28 (5.00-9.57) ng/dL and median prostate volume was 50.00 (36.92-65.00) mm3 in 252 patients.

No significant difference was found between the two groups with regard to age, BMI, serum PSA levels, and prostate volumes (p=0.612, p=0.966, p=0.615, p=0.627, respectively). However, the median number of cores and the malignancy detection rate were significantly higher in Group I compared to Group II (p <0.001, p=0.043, respectively) ( Table-1 ).

**Table 1:** Clinical characteristics of the patients in both groups.

	Group I (n=159)	Group II (n=93)	p
Age (years)	61.82 (±7.39)	62.28 (±6.16)	0.612
Body mass index (BMI) (kg/m^2^)	26.10 (22.80-27.70)	26.10 (22.80-28.30)	0.966
Total prostate volume (mm^3^)	51.62 (34.00-71.73)	50.00 (40.00-60.00)	0.627
Serum PSA level (ng/dL)	6.99 (5.01-10.10)	8.01 (5.05-9.12)	0.615
Number of biopsy cores (n)	16.0 (15.0-19.0)	12.0 (11.0-12.0)	<0.001
Malignancy detection rate (n, %)	70/159 (44.0%)	24/93 (25.8%)	0.004

**PSA** = Prostate specific antigen

No significant difference was found between the two groups with regard to VAS scores (p=0.070) ( Table-2 ). The most painful part of the whole procedure was revealed to be the insertion of the probe into the rectum ( Table-2 ). However, no significant difference was found between the groups with regard to the most painful biopsy step (p=0.140).

**Table 2:** Comparison of pain levels in both groups.

	Group 1 (n=159)	Group 2 (n=93)	p
VAS score	2.0 (1.0-4.0)	3.0 (1.0-5.0)	0.070
Most painful step			0.140
Probe insertion	111/159 (69.8%)	58/93 (62.4%)	
Probe manipulation	22/159 (13.8%)	22/93 (23.6%)	
Needle piercing	26/159 (16.4%)	13/93 (14.0%)	

**VAS** = Visual analog scale

## DISCUSSION

Our results suggested that FPB, when administered with an effective anesthetic technique, causes no extra pain compared to the standard biopsy techniques although it increases the number of biopsy cores and involves a relatively more complex procedure.

In a study conducted in 2018, Robins et al. reviewed 170 patients that underwent FPB or SPB and reported that no significant difference was found between the groups with regard to pain and discomfort ( 14 ). A previous prospective study by Arsoy et al. compared patient comfort between the patients that underwent MR-guided in-bore prostate biopsy and MRI/ultrasound fusion-guided prostate biopsy, in which PNB was induced by administering intrarectal anesthetic gel in all the patients, in a similar way to our study ( 15 ). The authors reported that FPB causes less pain compared to in-bore prostate biopsy although it increases the number of biopsy cores. In our study, the number of biopsy cores was higher in the FPB group compared to the SPB group. Despite the lack of supporting evidence, we consider that the acquisition of additional biopsy cores and the transfer of MpMRI images to the US fusion device are time-taking processes which lead to prolonged FPB procedures. However, despite these drawbacks of FPB, the pain levels experienced by the patients were revealed to be similar in both procedures.

Pain during transrectal prostate biopsy can be associated with the three steps of the biopsy procedure (i.e., insertion of the probe into the rectum, probe manipulation, and piercing of the biopsy needle) ( 16 ). In our study, we also divided the biopsy procedure into these three steps. Prior to the procedure, each patient was verbally informed about these steps and were asked to indicate the most painful step after the procedure. In both groups, the most painful step revealed to be the insertion of the probe into the rectum. A recent systematic review revealed that the administration of intrarectal anesthetic gel followed by PNB led to a lower degree of pain both during probe movements and needle piercing but had no remarkable effect on the pain experienced during the insertion of the probe ( 17 ). Urabe et al. compared the effectivity of intrarectal local anesthetic, PNB, and the combined methods in alleviating the pain during TRUS-guided SPB and reported that PNB led to lower pain levels during the insertion of the probe compared to other techniques ( 18 ). It should be noted that there are some studies in the literature which, in a similar way to our study, indicate that PNB alone or in combination with intrarectal gel leads to reduction in the pain experienced during probe manipulation and needle piercing ( 19 , 20 ).

Literature reviews also indicate that there is a controversy in the literature as to whether the level of pain during biopsy can vary according to patient age and prostate volume ( 21 - 23 ). In our study, we found that the mean age and the median prostate volume were similar in both groups (p=0.612, p=0.627, respectively), which is important for implicating standardization and homogeneity.

A recent systematic review indicated that the overall cancer detection rate was 26.3%-56.6% in SPB as opposed to 33.7%-79.5% in FPB ( 10 ). Similarly, Fourcade et al. reported that the overall cancer detection rate was higher in FPB compared to SPB (45% vs. 33.5%, p=0.02) ( 24 ). In our study, we found that the malignancy detection rate was significantly higher in FPB compared to SPB (44% vs. 25.8%, p=0.004). We consider that this difference was expected since the SPB group only comprised patients who were detected with a PI-RADS <3 lesion on MpMRI. On the other hand, it is also possible that the patients excluded from the study might have led to inconclusive or unrealistic results. Therefore, we consider that the oncological findings obtained in our study may not reflect the reality of the situation.

Our study was limited in several ways. First, our study had a relatively small patient population. Secondly, although the duration of FPB is known to be longer than that of SPB, we did not record the durations of the procedures and thus could not evaluate the association between the duration of the procedure and pain. Thirdly, we did not assess the VAS scores separately for each of the three steps of the procedure (probe insertion, probe manipulation, and needle piercing) and only asked the patients to indicate the most painful step after the procedure. As a result, we could not perform an objective evaluation on the biopsy steps. Finally, the location of the suspected lesion may be important for pain felt during the biopsy. In particular, sampling from the anterior region or central zone may cause more pain as it is more difficult to reach. Another limitation of this study was the lack of statistical comparison on subject of lesion localization/pain levels due to insufficient number of patients.

## CONCLUSIONS

Despite that MRI-US fusion and taking additional cores prolonged the predicted duration of FPB which has higher cancer detection rate comparing with SPB, pain level during FPB was similar to SPB. We consider that these drawbacks of FPB do not have any adverse effects on pain. Further prospective studies with larger patient series are needed to substantiate our findings.
